# PPARG Pro12Ala Polymorphism with CKD in Asians: A Meta-Analysis Combined with a Case-Control Study—A Key for Reaching Null Association

**DOI:** 10.3390/genes11060705

**Published:** 2020-06-26

**Authors:** Hsiang-Cheng Chen, Wei-Teing Chen, Tzu-Ling Sung, Dung-Jang Tsai, Chin Lin, Hao Su, Yuh-Feng Lin, Hung-Yi Chiu, Sui-Lung Su

**Affiliations:** 1 Division of Rheumatology/Immunology/Allergy, Department of Internal Medicine, Tri Service, General Hospital, National Defense Medical Centre, Taipei 11490, Taiwan; hccheng@ndmctsgh.edu.tw; 2 Division of Chest Medicine, Department of Medicine, Cheng Hsin General Hospital, Taipei 11220, Taiwan; unirigin@gmail.com; 3 Department of Medicine, Tri-Service General Hospital, National Defense Medical Center, Taipei 11490, Taiwan; 4 School of Public Health, National Defense Medical Center, Taipei 11490, Taiwan; friend2158251@gmail.com (T.-L.S.); oo800217@gmail.com (D.-J.T.); xup6fup0629@gmail.com (C.L.); 5 Graduate Institute of Life Sciences, National Defense Medical Center, Taipei 11490, Taiwan; 6 Department of Health Industry Management, Kainan University, Taoyuan 33857, Taiwan; d131419@gmail.com; 7 Division on Nephrology, Department of Internal Medicine, School of Medicine, College of Medicine, Taipei Medical University, Taipei 11031, Taiwan; 8 Division of Nephrology, Department of Medicine, Tri-Service General Hospital, National Defense Medical Center, Taipei 11490, Taiwan; 9 School of Public Health, Taipei Medical University, Taipei 11490, Taiwan

**Keywords:** chronic kidney disease, peroxisome proliferator-activated receptor, gene polymorphism, case-control study, meta-analysis, trial sequential analysis, meta-regression, gene-environment interaction

## Abstract

Background: So far, numerous meta-analyses have been published regarding the correlation between peroxisome proliferator-activated receptor gamma (PPARG) proline 12 alanine (Pro12Ala) gene polymorphism and chronic kidney disease (CKD); however, the results appear to be contradictory. Hence, this study is formulated with the objective of using existing meta-analysis data together with our research population to study the correlation between PPARG Pro12Ala gene polymorphism and CKD and evaluate whether an accurate result can be obtained. Methods: First, literature related to CKD and PPARG Pro12Ala available on the PubMed and EMBASE databases up to December 2016 was gathered from 20 publications. Then, the gathered results were combined with our case-control study of 1693 enrolled subjects and a trial sequential analysis (TSA) was performed to verify existing evidence and determine whether a firm conclusion can be drawn. Results: The TSA results showed that the cumulative sample size for the Asian sample was 6078 and was sufficient to support a definite result. The results of this study confirmed that there is no obvious correlation between PPARG Pro12Ala and CKD for Asians (OR = 0.82 (95% CI = 0.66–1.02), I^2^ = 63.1%), but this was not confirmed for Caucasians. Furthermore, the case-control sample in our study was shown to be the key for reaching this conclusion. Conclusions: The meta-analysis results of this study suggest no significant correlation between PPARG Pro12Ala gene polymorphism and CKD for Asians after adding our samples, but not for Caucasian.

## 1. Introduction

Examining the risk factors of chronic kidney disease (CKD) is an important public health issue. The global prevalence of CKD is around 10% [1,2,3], and such patients are at a high risk of developing cardiovascular diseases or even death [4]. Genetic factors such as ethnic origin [5] and family inheritance [6] play an important role in the progression of CKD. The heritability of CKD is 20–80%, and individual variations are due to genetic variations [7,8,9,10]. Earlier genome-wide association studies have identified many gene segments that may affect the risk of developing CKD [11,12]. Therefore, studying CKD-associated genes is a crucial step toward decreasing the risk of contracting the disease.

One of the factors that can affect CKD, as well as insulin resistance, is the functioning of peroxisome proliferator-activated receptor gamma (PPARG). PPARG is a transcription factor that regulates the differentiation and proliferation of adipose cells and the metabolism of fatty acids and carbohydrates [13]. The functions of PPARG include alleviating inflammatory factors, increasing the glucose use rate in muscles, and decreasing liver gluconeogenesis, thereby affecting insulin sensitivity [14,15]. Therefore, PPARG activation is commonly prescribed in blood glucose-lowering medications (such as thiazolidinediones) because it can promote the secretion of adiponectin and lipid-lowering proteins by adipocytes and inhibit inflammatory responses at the same time, thereby increasing insulin sensitivity in muscle tissues [16]. Beside diabetes treatments, PPARG activation decreases kidney fibrosis and inflammation and reduces retention of salt and water in renal tubules to prevent matrix expansion, thereby alleviating damage to kidney cells [17]. In short, the dysregulation of PPARG can cause insulin resistance and CKD.

There are two isoforms of PPARG: PPARG1 and PPARG2; both isoforms are produced by alternative splicing. There are 28 more amino acids at the amino terminus of PPARG2, and the efficiency of this domain is 5–10 times that of PPARG1 [18]. Both PPARG1 and PPARG2 are highly expressed in adipose tissues, but PPARG1 is also found in the liver, spleen, and heart [19]. A previous animal study found that rats with a low level of PPARG2 mRNA expression in adipose tissues do not develop obesity or insulin resistance even after being given a high calorie diet [20]. Because insulin resistance activates protein kinase C (PKC) and promotes the secretion of transforming growth factor-beta (TGF-β) by glomerular mesangial cells, the expression of PPARG2 may cause the accumulation of microvascular matrix proteins and microvascular obstruction, thereby increasing the risk of nephropathy [21,22,23]. Hence, the functions of PPARG2 may be associated with the risk of CKD, and PPARG polymorphisms may affect this risk.

Proline 12 alanine (Pro12Ala) is a single-nucleotide polymorphism (SNP) located on the exon B of the PPARG gene. When SNP is a cysteine (C), the amino acid would be a proline (Pro subtype), and when SNP is a guanine (G), the amino acid will be an alanine (Ala subtype) [24]. Previous studies have indicated that in the Ala subtype, the ligand affinity of the PPARG2/retinoid X receptor (RXR) heterodimer is lower than that in the Pro subtype. The affinity to peroxisome proliferator response elements (PPREs) in the Ala subtype is 1.6–2.5 times lower than that in the Pro subtype; the ability of activating target transcription is also poorer in the Ala subtype, resulting in a reduced accumulation of adipose tissue, which improves insulin sensitivity. Therefore, the PPARG2/RXR heterodimer in the Ala subtype can reduce an individual’s body mass index (BMI) and improve insulin resistance [18]. Hence, the G allele in PPARG Pro12Ala may be a protective factor against CKD.

Numerous publications have discussed the correlation between PPARG Pro12Ala and CKD; the results show that compared with the C/C genotype, the G/G or G/C genotype of PPARG Pro12Ala is a protective factor against diabetic nephropathy [25,26,27,28,29,30] or CKD [31]. However, there is one study suggesting an opposite conclusion [32]. Hence, the correlation between PPARG Pro12Ala and CKD remains controversial.

Earlier meta-analyses examining the relationship between PPARG Pro12Ala and CKD found that the G/G and G/C genotypes are protective factors against CKD [33,34,35,36,37,38,39]. However, no definite conclusion can be drawn based on these meta-analyses [33,34,35,36,37,38,39] because (1) previous meta-analyses assumed a dominant model (G/G or G/C genotype compared with the C/C genotype), although it is still not proven that the model is the most appropriate inheritance mode for Pro12Ala and (2) artificial errors may occur when the model selection for combining data is based on the degree of heterogeneity [40]. In addition, there are new studies published after 2013 (including 2321 patients) that have not been included in any meta-analysis [41,42,43,44,45,46]; hence, the existing meta-analyses are unable to provide a definite conclusion based on current evidence.

Following the current state-of-art, this study is formulated with the objective to alleviate the heterogeneity between different studies and attain a constructive conclusion regarding the relationship between PPARG Pro12Ala and CKD. For this purpose, a case-control study performed by the authors was combined with a meta-analysis dataset to examine the correlation between PPARG Pro12Ala and CKD and investigate the effects of the interaction between environmental factors and PPARG Pro12Ala on CKD. In addition, trial sequential analysis (TSA) was used to show that a definite conclusion can be drawn from the meta-analysis.

## 2. Materials and Methods

### 2.1. Meta-Analysis

#### 2.1.1. Search Methods and Criteria for Study Consideration

The first step in conducting a meta-analysis is the careful selection of reporting items. A checklist of preferred reporting items for systematic reviews and meta-analyses and meta-analysis on genetic association studies is provided in Table S1 [47]. The sampling group considered in this study was the general population, and the aim was to investigate the correlation between CKD risk and individuals carrying the major (C) and minor (G) alleles of PPARG Pro12Ala. A search of the PubMed and EMBASE databases was carried out, based on synonyms for “PPARG Pro12Ala” and “CKD” for publications written in English and published before 31 December 2016 (details are shown in Table S2). The publications cited in previous meta-analyses were also manually inspected to avoid missing crucial references.

The inclusion criteria for publications in our meta-analysis were as follows: (1) the study was a case-control study or a cross-sectional study; (2) the case group showed a definite diagnosis of renal function abnormalities, such as proteinuria, high serum creatinine, and low glomerular filtration rate, or damages to the kidney structure, excluding lupus nephritis, polycystic kidney disease, endemic nephropathy, and reflux nephropathy, based on kidney biopsy, CT scan, or ultrasound; (3) the patients in the control group had normal renal function; (4) the publication provided the allelic distribution for the target locus; and (5) the sample comprised only adults aged over 18 years. The publications containing incomplete genetic information were excluded. To alleviate the heterogeneity across different studies, when the case group in a study contained 100% of diabetics, data was extracted from the control group with 100% of diabetics as well.

#### 2.1.2. Data Extraction

Two reviewers (Tzu-Ling Sung and Chin Lin) were employed to independently extract targeted information from the literature. The extracted information included the last name of the first author, year of publication, country, and ethnicity of the study population, sex ratio, mean BMI, prevalence of diabetes, and allelic distribution of the targeted gene in the case and control groups.

#### 2.1.3. Statistical Analysis

All selected publications were described using appropriate ratios or mean values. In this meta-analysis, odds ratios (ORs) were used with 95% confidence intervals (CIs). The I^2^ statistic—calculated using the Cochrane Q test—was used to evaluate the heterogeneity among published studies. A result of I^2^ > 50% was considered to indicate moderate-to-high heterogeneity [48]. Egger’s regression and a funnel plot were used to examine the symmetry after combination. Genetic models, including allele type, and dominant and recessive models were used to calculate PPARG Pro12Ala polymorphisms and CKD risk, and a random-effects model was used to combine the results. Meta-regression was used to inspect the source of heterogeneity.

Possible environmental risk factors such as ethnic origin, sex, BMI, and diabetes, were also included in the analysis and this data were extracted from the case group to investigate the interactions between genes and the environment [49,50]. The “metafor” [51] and “meta” [52] packages in R software version 3.3.1 (R Project for Statistical Computing, Vienna, Austria) were used for statistical analyses, and the significance level was set at 0.05. TSA was performed in order to verify whether the meta-analysis result was a definite conclusion [53]; ORs were used as effect measures, and the random-effects model was used to combine results. Zero event handing was set as 1, CI was set as 95%, and the number of degrees of freedom was set as 2. TSA also involved stratified analysis based on ethnic origin (i.e., Caucasian vs. Asian). The number of patients with the G and C allele was input for the case group and control group, respectively. For the event, the number of CKD patients was entered, and for the total, the total number of people in the CKD and healthy groups was input. The type 1 error was set as 0.05 and the power was set as 0.8 for sample estimation. For the heterogeneity test, the internal TSA diversity value (Caucasian: 76%, Asian: 71%) was input. An OR value of 1.5 can be considered a reasonable number for establishing a correlation between genes and disease [54]; however, because the G allele could be a protective factor here, the OR value was set as the reciprocal of 1.5 (i.e., OR = 0.67). The minor allele frequency referred to the 1000 genome database: 0.03 and 0.12 for Asians and Caucasians, respectively [55].

### 2.2. Case-Control Study

#### 2.2.1. Ethical Issues

This study was approved by the institutional review board (TSGH-1-104-05-006) of the Tri-Service General Hospital (TSGH), a medical teaching hospital of the National Defense Medical Centre in Taipei, Taiwan. All volunteers had signed the informed consent form after being provided with an explanation by the investigator.

#### 2.2.2. Subjects

Patients in the case group were selected from hemodialysis patients admitted in the dialysis centers or clinics in Taipei between 2014 and 2015 after diagnosis by professional physicians. The following patients were excluded: (1) those with a dialysis period of less than three months, (2) those with cancer, and (3) those from whom insufficient blood samples were obtained. Finally, 847 patients were enrolled in the case group for analysis. The control group comprised volunteers who participated in health examination in the Health Management Center of TSGH.

The serum creatinine of the volunteers was measured and the eGFR values were calculated using the Modification of Diet in Renal Disease equation with adjustments for sex and age [56]. The following patients were excluded from the study: (1) those with an eGFR < 60 mL/min/1.73 m^2^, (2) those with kidney-related disorders (such as positive proteinuria), (3) those with cancer, and (4) those from whom insufficient blood samples were obtained. Eventually, 846 volunteers were enrolled in the control group for the analysis.

Demographic data such as age, sex, diabetes, hypertension, BMI (kg/m^2^), and blood biochemical values (blood urea nitrogen, creatinine, fasting blood glucose, triglycerides, cholesterol, and eGFR) were collected from questionnaires and medical records.

#### 2.2.3. Genomic DNA Extraction and Genotyping

A 5-mL blood sample was intravenously extracted from volunteers by physicians or nurses. The genomic DNA was isolated from peripheral blood samples with standard procedures for proteinase K (Invitrogen, Carlsbad, CA, USA) digestion and phenol/chloroform methods [57]. The PPARG Pro12Ala locus was genotyped using the iPLEX Gold SNP genotyping method [58]. Inter- and intra-replication validation was used to assess the quality of the genotyping experiment. Inter-replication validation was repeated for 78 samples (about 5%), and the concordance rate was 100%. Ten samples were randomly selected and intra-replication validation was performed using polymerase chain reaction according to a previously described protocol [59]. A second genotyping was performed, and the results were compared with the results of the first one: the concordance rate between the two genotyping methods was 100%.

#### 2.2.4. Statistical Analysis

The continuous variables for the demographic data were shown as a series of means ± standard deviations and tested using the Student’s *t*-test. The Hardy–Weinberg equilibrium [60] was used to test the representativeness of the control group. The differences in genotypes and allelic frequencies of hemodialysis patients and healthy controls were tested using χ^2^ test or Fisher’s exact test. The OR and 95% CI for the risk of kidney failure (ESRD) was calculated using logistic regression. The calculation of the risk between genetic polymorphism and ESRD was represented by the allele type, genotype, and dominant/recessive models. Statistical significance was set as *p* < 0.05 after the Bonferroni correction. As in the case of meta-analysis, the statistical analyses were conducted using R software version 3.3.1(R Foundation for Statistical Computing, Vienna, Austria).

## 3. Results

### 3.1. Meta-Analysis

**Figure 1:**
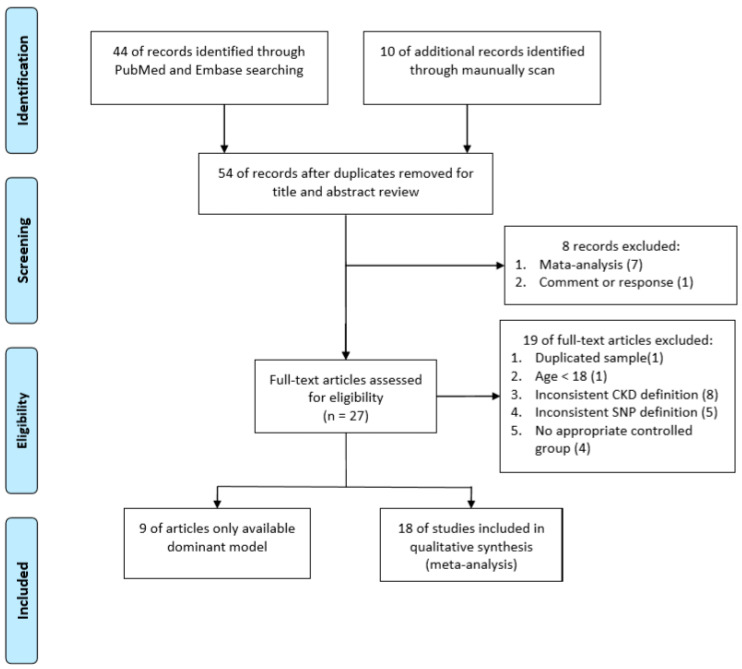
Flow diagram for the identification process of eligible studies.

The search flowchart is shown in Figure 1. For the meta-analysis, 44 publications were found in the PubMed and EMBASE databases, as well as 10 publications cited in previous meta-analysis studies that were missing from the databases, thereby obtaining a total of 54 publications for further analyses. Based on the topic and abstract, seven meta-analyses and one review article were excluded. Based on the main text, two publications that used the same dataset, one publication with non-adult samples, eight publications with incompatible definitions of case groups, five publications that focused on different genes, and three non-case-control studies were also excluded. Because the study conducted by De Cosmo [39] provided three samples, finally, there were 29 samples included in the meta-analysis (details shown in Table S3). Among these, 20 studies provided complete genotypes (C/C, C/G, and G/G genotypes) and nine studies provided only the dominant model (C/G or comparison between G/G and C/C genotypes), for details see Table S4.

Herein, we used the dominant model to combine the 29 samples, and the result was statistically significant (OR = 0.76 (95% CI = 0.63–0.92)); the details are shown in Table 1 (forest plot and funnel plot are shown in Figure S1). In particular, the result of combining the nine samples with only dominant genotype data was statistically significant (OR = 0.69 (95% CI = 0.50–0.95)); however, the result of combining the 20 samples with complete genotype data was not statistically significant (OR = 0.80 (95% CI = 0.62–1.03)). Therefore, there could potentially be some publication bias among publications that only provided dominant genotypes, and the bias may be due to the exclusive use of Caucasian samples (Egger’s test, *p* = 0.032). In this study, the allele model was used to combine results of 20 samples, and the result was not statistically significant (OR = 0.82 (95% CI = 0.65–1.04)). The heterogeneity (I^2^) was 63.1%, and there was no significant asymmetry found among the studies (the forest plot and funnel plot are shown in Figure S2).

### 3.2. TSA Sample Estimation

**Figure 2:**
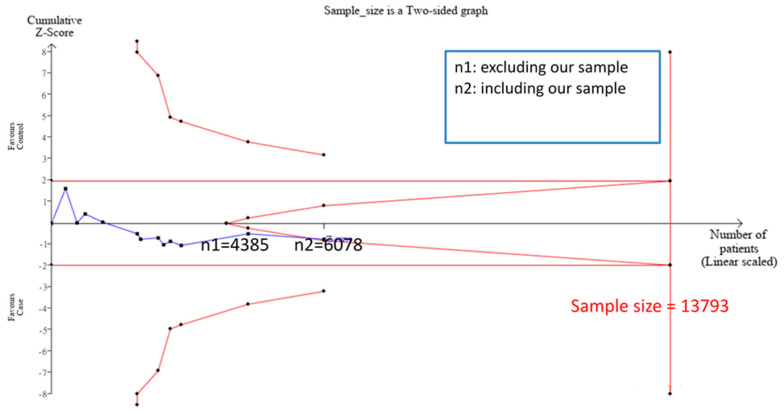
Trial sequential analysis (TSA) used in this meta-analysis for the Asian sample. TSA is a methodology that includes a sample size calculation for a meta-analysis with the threshold of statistical significance. TSA was performed using an allele model assumption but replacing the allele count with the sample size (divided by 2). Detailed settings: Significance level = 0.05; power = 0.80; ratio of controls to cases = 1; hypothetical proportion of D allele in control = 0.03; least extreme OR to be detected = 0.67; I^2^ (heterogeneity) = 71%.

TSA estimation showed that the cumulative sample size for Caucasians was 2428 (Figure S3), and there was no significant correlation between PPARG Pro12Ala and CKD (with the allele model). However, the cumulative sample size was below the targeted sample size (*n* = 13,002); therefore, no definite conclusion could be drawn from the meta-analysis for the Caucasian sample. The cumulative sample size for the Asian sample was 4385 (Figure 2), and there was no significant correlation between PPARG Pro12Ala and CKD (with the allele model). The cumulative sample size for the Asian sample was below the targeted sample size (*n* = 13,793); therefore, no definite conclusion could be made from the meta-analysis for the Asian sample as well. Nevertheless, the cumulative sample size for the Asian sample appeared to be approaching the futility area (Figure 2). Therefore, adding samples from our case-control study (*n* = 1693) into the meta-analysis for further examination was justified.

### 3.3. Case-Control Study

The distribution of demographic variables and blood biochemical values for the study population is shown in Table 2. A total of 1693 subjects was enrolled in our study, including 846 volunteers for the control group with a mean age of 73.50 ± 7.21 years (377 males and 469 females) and 847 patients for the case group with a mean age of 71.84 ± 12.93 years (430 males and 417 females). The male ratio in the control group was lower than in the case group (*p* = 0.011), and the mean age in the control group was higher than in the case group (*p* = 0.001). Compared with the control group, the case group showed a higher ratio of diabetes and hypertension and higher levels of urea nitrogen, creatinine, preprandial blood glucose, and triglycerides. In addition, the case group showed a lower cholesterol and glomerular filtration rate. The difference in the genetic distribution of PPARG Pro12Ala between the case and control groups is shown in Table 3. The gene frequency in the control group was consistent with the Hardy–Weinberg equilibrium (*p* = 0.491). The distribution of the G allele in the control and case groups was 94.8% and 5.2%, respectively, but the difference was not statistically significant (*p* = 0.083). Similarly, there was no significant correlation between PPARG Pro12Ala and ESRD [OR = 0.75 (95% CI = 0.54–1.04)] (Table 4). We further used the genotype and dominant/recessive models to validate the results and found no significant differences. Therefore, the data from our case-control study indicated that there was no significant correlation between PPARG Pro12Ala and ESRD. Hence, as stated previously, in order to increase the evidence level of the results, the case-control study sample was added to the meta-analysis and TSA was used to verify if a definite conclusion could be drawn.

### 3.4. Meta-Analysis Results after Addition of Case-Control Study and TSA Sample Estimation

**Figure 3:**
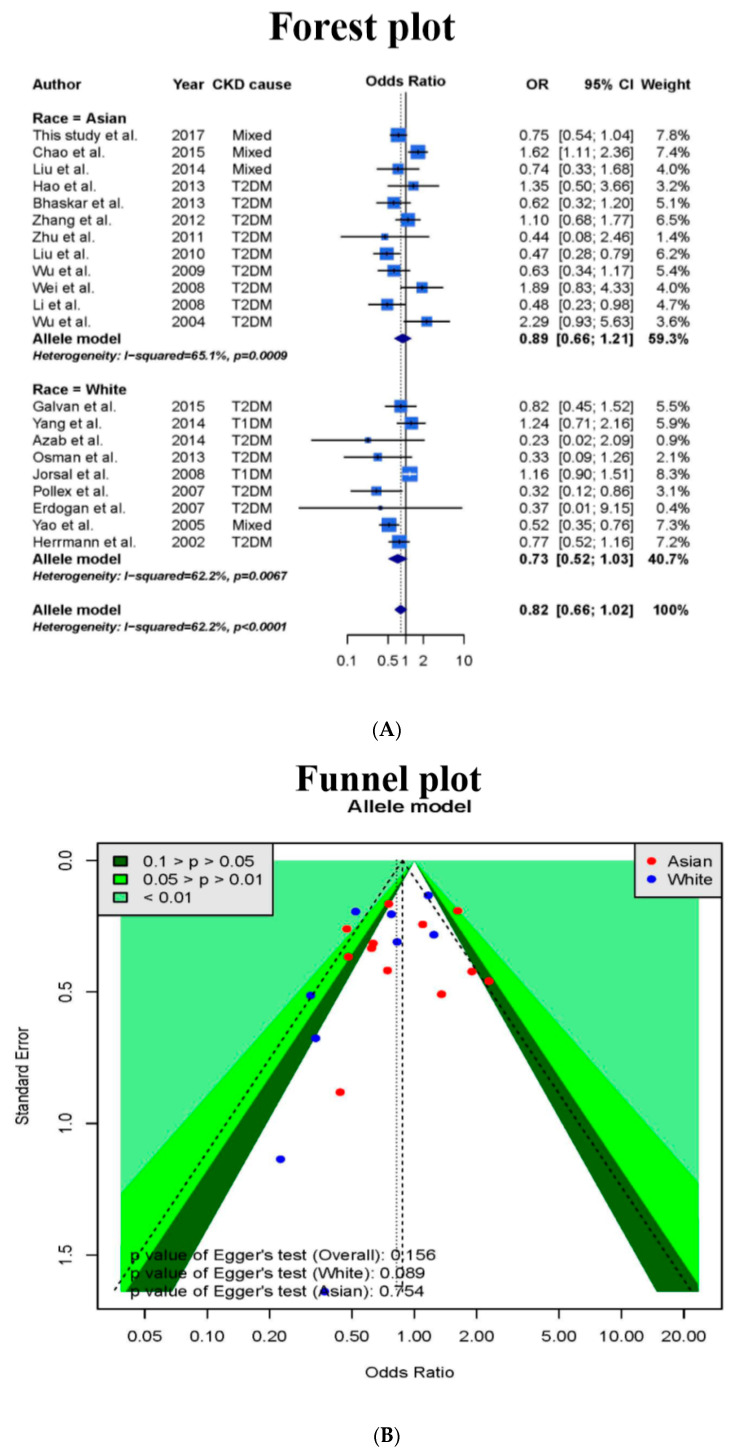
Forest plot and funnel plot showing the correlation between PPARG Pro12Ala alleles and CKD (including this study). (**A**) Forest plot of allele comparison for overall comparison. The list of article references are in Supplementary Table S5. (**B**) Funnel plot analysis showed no potential publication bias.

After adding the case-control study sample, the allele model was used to combine the 21 samples (20 + 1) and an insignificant result was found (OR = 0.82 (95% CI = 0.66–1.02), I^2^ = 63.1%). There was no potential publication bias (the forest and funnel plots are shown in Figure 3). In addition, in the stratified analysis based on the ethnic origin, the results for the Asian as well as Caucasian samples were not significant (OR = 0.89 (95% CI = 0.66–1.21), I^2^ = 65.1% and OR = 0.73 (95% CI = 0.52–1.03), I^2^ = 62.2%, respectively). We further used the dominant/recessive model to combine the data, but the result was still not significant (Figure S4). By including the sample from our case-control study (n = 1693), TSA was used to estimate the sample size for the Asian sample (Figure 2) and it was found that the cumulative sample size was 6078 with an insignificant correlation between PPARG Pro12Ala and CKD (with the allele model). The cumulative sample size (the Z curve) was in the futility area; therefore, it can be concluded that n = 6078 was sufficient to support a definite result. This study confirmed that for Asians, there is no obvious correlation between PPARG Pro12Ala and CKD (with the allele model), and our study sample was the key sample to ascertain the conclusion.

### 3.5. Interactions between the Gene and Environmental Factors

Because the heterogeneity could still be detected in the meta-analysis (as shown in Figure 3), it is possible that the correlation between PPARG Pro12Ala and CKD is affected by some environmental factors. The effects of regulatory factors in the meta-analysis of PPARG Pro12Ala and CKD (with the allele model) are shown in Table 5. We used meta-regression analysis to examine the environmental effects of ethnic origin, sex, BMI, and diabetes. Without adjusting other regulatory factors, no environmental factor was found to affect the relationship between PPARG Pro12Ala and CKD (*p* > 0.0125 for all factors, after Bonferroni correction). Hence, it was confirmed that there was no correlation between PPARG Pro12Ala and CKD (with the allele model) and no environmental factors interacted with PPARG Pro12Ala and CKD.

## 4. Discussion

The obtained results suggested that there is no significant correlation between PPARG Pro12Ala and CKD, and the same result was observed in both the case-control study and meta-analysis, including 21 samples. Following this, it can be hypothesized that compared with the C allele in the PPARG Pro12Ala, the G allele has poorer affinity between the PPARG2/RXR heterodimer and its ligand, decreasing the transcriptional ability of the target gene, resulting in decreased adipose tissue accumulation, and improving insulin sensitivity [18]. Insulin resistance increases the risk of proteinuria and causes kidney deterioration, thereby resulting in nephropathy [21,22,23]. If insulin sensitivity is improved, the possibility of developing nephropathy can be reduced [17]. Based on this mechanism, the G allele of PPARG Pro12Ala should be a protective factor for CKD; however, our results show a negative finding and contradict this hypothesis.

One possible cause underlying such a negative finding may be the existence of other undetected factors that affect the correlation between PPARG Pro12Ala and CKD, such as the effect of ligand on PPARG functioning [38]. PPARG is a transcription factor that binds to RXR and forms a heterodimer, which further binds to PPREs on the target gene promoter after being activated by a ligand and promotes the transcription of target genes (such as lipoprotein lipase, and uncoupling protein 2) [13]. The mechanism regulates the differentiation and proliferation of adipose cells, and the metabolism of fatty acids and carbohydrates [12]. The source of ligands includes synthetic ligands (e.g., PPARG agonists, blood lipid-lowering drugs, insulin sensitizers) and endogenous ligands (e.g., fatty acids or other derivatives) [61].

In mouse experiments [20], the mice fed with a high calorie diet (ligand stimulation) show lower PPARG concentrations in their adipose tissues, indicating a significant protective effect pertaining to insulin resistance. At the same time, the mice fed with a normal diet (no ligand stimulation) show no significant difference in insulin resistance, regardless of PPARG concentrations in their adipose tissues. A previous study has shown that under ligand stimulation, the Ala subtype of PPARG2/RXR heterodimer lessens the functioning of PPARG2 in adipose tissues, thereby decreasing the levels of fat accumulation and resulting in a lower risk of insulin resistance; however, under conditions with no ligand stimulation, there were no significant differences between the Pro and Ala subtype heterodimers in fat accumulation or insulin resistance [18]. The cited study suggested that for patients with the C/C genotype in PPARG Pro12Ala, serum triglyceride concentrations are higher than those for patients with the G/G genotype; however, if a diet of polyunsaturated fatty acids is given to patients with the C/C genotype (i.e., a reduced ligand stimulation), the serum triglyceride concentrations can be significantly reduced [62]. Accordingly, it can be inferred that different dietary intake (i.e., the activation of ligand stimulation) may affect PPARG function and influence the risk of CKD, and ligand stimulation could be the cause of the negative finding observed in this study.

It is the authors’ opinion that different ethnic origins may have different effects on the correlation between PPARG Pro12Ala and CKD. Our study found that there was no significant correlation between PPARG Pro12Ala and CKD in the Caucasian sample, whereas results in previous meta-analyses indicated that the PPARG Pro12Ala dominant model has a significant protective effect against CKD [32,33,34,37,38,39]. One possible explanation for this is that our study excluded publications with incomplete genetic information, and precisely these excluded studies suggested that PPARG Pro12Ala has a significant protective effect against CKD (Table 1).

We suspect that the exclusion resulted in a tendency of including insignificant results. Moreover, the Caucasian sample size used in this study was insufficient according to TSA; therefore, the meta-analysis results were not definite. Although there could potentially be publication biases, the sample size of previous meta-analyses was relatively sufficient compared with that of our study. Hence, the Caucasian PPARG Pro12Ala dominant model may have protective effects against CKD. In addition, our case-control study found that there is no significant correlation between PPARG Pro12Ala and ESRD; this is consistent with the findings published in most previous observations pertaining to Asian populations [27,38,44,45,63,64,65,66]. To emphasize the level of evidence, our meta-analysis also indicated no significant correlation between PPARG Pro12Ala and CKD in the Asian sample; this was consistent with the results of previous meta-analyses for Asians [33,34,36,37,38]. However, no definite conclusions could be made from previous meta-analyses for Asians; therefore, TSA was used on our study to determine whether a definite conclusion can be obtained [67]. As shown earlier, after adding the 1693 samples from our case-control study, the cumulative sample size for the Asian sample entered the futility area defined by TSA (Figure 2), enabling 95% confidence to declare that there is no significant correlation between PPARG Pro12Ala and CKD. Therefore, we can suspect that PPARG Pro12Ala has protective effects against CKD in Caucasians but not in Asians.

The ligand could be responsible for the difference between ethnic groups. It is possible that the environmental factors or ligands in Asians have some modifying effects on PPARG Pro12Ala and CKD [38]. The amount of high calorie food consumed by Asians is relatively lower than that by Caucasians [68], and high calorie food is the main ligand for PPARG [61]. Therefore, the protective effect of PPARG Pro12Ala against CKD in Asians may be masked.

To identify the source of heterogeneity among publications, we further examined the effects of environmental factors on PPARG Pro12Ala and risk of CKD. Results from previous studies have shown that the G/G or G/C genotype has a protective effect against diabetic nephropathy in Caucasians but not in Asians, and it is known that ethnic origin is an important regulatory factor [37,38]. The proportion of diabetics may be a modifying factor because the PPARG Pro12Ala G carrier would have a significant protective effect against CKD when the proportion of CKD patients with diabetes was 100%, and no significant difference was observed when the proportion of diabetics decreased [44]. In addition, male subjects with the PPARG Pro12Ala G/G or G/C genotype are found to have higher BMI, and sex has a significant interaction effect (*p* = 0.039) on Pro12Ala and BMI (*p* = 0.039) [69]. Subjects showing the PPARG Pro12Ala C/C genotype with a BMI > 30 kg/m^2^ tend to have higher blood glucose values and insulin resistance; thus, BMI is potentially a modifying factor in the interaction [70]. In our study, a retrospective analysis was used to examine the effects of ethnic origin, sex, BMI, and proportion of diabetics on PPARG Pro12Ala and CKD but it was found that these environmental modifying factors do not affect the correlation between PPARG Pro12Ala and CKD (Table 5). As no gene–environment interactions were found, further examination is needed to identify the source of heterogeneity.

It can be hypothesized that the source of heterogeneity lies in the SNP variations on the PPARG binding site or gene–gene interactions. The targeted gene can only be transcribed after the PPARG/RXR is bound to specific regulatory sequences (PPREs) [14]. A previous study indicated that the transcription of hepatic low-density lipoprotein receptor genes is regulated through the variations in PPARG PPREs [71]; hence, SNP variations in PPARG PPREs may prevent heterodimer binding and thereby affect downstream gene transcription. SNP variations may occur in specific ethnic groups, resulting in differences in results between ethnic groups and a high level of heterogeneity between studies. Furthermore, SNP variations on the binding site and PPARG Pro12Ala gene–gene interactions may also affect PPARG functioning. To fully explain the source of heterogeneity between studies, future investigations should further examine the effects of SNP variations in the PPARG binding site on CKD or the interactions between the SNP on the binding site and Pro12Ala (i.e., gene-gene interactions).

Compared with previous studies, our meta-analysis appears to reduce artificial errors. Publications with incomplete genotypes were excluded and the allele model was used to combine results, thereby avoiding publication selecting bias due to the use of the dominant model (Table 1). Our meta-analysis suggested that Egger’s regression is not significant (*p* ≥ 0.05), neither for Asian nor Caucasian samples, and the results from literature were balanced (Figure 3). Previous meta-analyses used the fixed effect model to combine results when the heterogeneity was not significant (*p* ≥ 0.1) but used the random-effect model when the heterogeneity was significant (*p* < 0.1) [33,34,35,36,37,38,39]. In our study, the random-effect model was used to combine all results and avoid serious errors caused by selection of models according to the degree of heterogeneity [40].

Finally, our study has some limitations. First, this was a meta-analysis with no information on individual medication and dietary status. This may lead to overlooking the effects of ligands and underestimating the correlation between PPARG Pro12Ala and CKD. Second, some studies did not supply a comprehensive distribution for the regulatory factors (e.g., sex, BMI, and proportion of diabetes). Third, the roles of PPAR gamma in lipid-energy metabolism might be associated with metabolic syndrome. However, the literature included in this study does not contain data on metabolic syndrome. Therefore, meta-regression cannot be performed on metabolic syndrome; therefore, the data used in this study was not always complete. Nevertheless, a meta-analysis was performed, combining further case-control study samples that should sufficiently increase the level of evidence and a meta-regression was carried out that examined the interaction between the gene and environment. In addition, TSA was used to test the results of the meta-analysis and it was possible to draw a definite conclusion, despite some data being incomplete.

## 5. Conclusions

In this study, a meta-analysis of literature related to CKD and PPARG Pro12Ala was performed along with our case-controlled study of 1693 enrolled subjects. TSA was used to verify the conclusions of the meta-analysis and it was found that for Asians, the PPARG Pro12Ala gene polymorphism does not have a significant correlation with CKD, and there is no gene-environment interaction. At the same time, definite results could not be obtained for Caucasians because of insufficient sample size. Heterogeneity, nevertheless, exists in this study; future studies should focus on in-depth examination to determine whether the SNPs in PPREs would affect the correlation between PPARG and CKD and to examine the gene-gene interactions of SNPs in PPREs and Pro12Ala.

## Figures and Tables

**Table 1:** Combined results of the dominant model (GG + GC vs. CC) for examining the correlation between PPARG Pro12Ala and CKD.

Model	Race	*n*	Sample Size	Odds Ratio (95% CI)	*I* ^2^	Egger’s Test
All	All studies	29	13,340	0.76 (0.63–0.92)	61.6%	0.065
	Asian	12	6017	0.92 (0.67–1.26)	60.8%	0.354
	Caucasian	17	7323	0.67 (0.52–0.86)	60.8%	0.032 *
Incomplete genotype	All studies	9	6527	0.69 (0.50–0.95)	63.9%	0.262
	Asian	1	1632	1.13 (0.69–1.84)	NA	NA
	Caucasian	8	4895	0.64 (0.46–0.91)	63.0%	0.294
Complete genotype	All studies	20	6813	0.80 (0.62–1.03)	61.3%	0.100
	Asian	11	4385	0.90 (0.62–1.28)	63.7%	0.411
	Caucasian	9	2428	0.69 (0.48–1.01)	61.3%	0.087

*: *p*-value of Egger’s regression < 0.05. GG and CC: homozygous genotype of PPARG Pro12Ala. GC: heterozygous genotype of PPARG Pro12Ala.

**Table 2:** Demographic and blood biochemical values distribution for ESRD patients.

Dependent Variable Independent Variable	Control ^a^ (*n* = 846)	ESRD ^b^ (*n* = 847)	*p*-Value
Male, (%)	377 (44.6%)	430 (50.8%)	0.011 *
Age, (mean ± SD, years)	73.50 ± 7.21	71.84 ± 12.93	0.001 *
Diabetes, (%)	105 (12.5%)	372 (80.3%)	<0.001 *
Hypertension, (%)	358 (42.7%)	222 (81.3%)	<0.001 *
BMI, (mean ± SD), kg/m^2^	24.22 ± 3.37	24.66 ± 4.80	0.420
Blood biochemistry values (mean ± SD)			
Urea nitrogen, mg/dL	15.90 ± 3.89	73.88 ± 24.72	<0.001 *
Creatinine, mg/dL	0.83 ± 0.72	9.42 ± 2.78	<0.001 *
Preprandial blood glucose, mg/dL	102.48 ± 25.20	148.90 ± 75.40	<0.001 *
Triglycerides, mg/dL	103.62 ± 40.82	157.02 ± 98.75	<0.001 *
Cholesterol, mg/dL	185.05 ± 33.35	162.42 ± 45.16	<0.001 *
Glomerular filtration rate, mL/min/1.73 m^2^	93.81 ± 23.76	5.73 ± 2.45	<0.001 *

^a^: In the control group, eGFR > 60 mL/min/1.73 m^2^; ^b^: In the case group with hemodialysis patients, eGFR < 15 mL/min/1.73 m^2;^ * *p* < 0.05. ESRD = End stage renal disease

**Table 3:** PPARG Pro12Ala genotype distribution in ESRD case and control groups.

Genotype	Control ^a^ (*n* = 846)	ESRD Patients (*n* = 847)	*p*-Value
Alleles			0.083
C Allele	1604 (94.8%)	1627 (96.0%)	
G Allele	88 (5.2%)	67 (4.0%)	
Co-dominant			0.169
CC	762 (90.1%)	781 (92.2%)	
CG	80 (9.5%)	65 (7.7%)	
GG	4 (0.5%)	1 (0.1%)	
Dominant Model			0.122
CC	762 (90.1%)	781 (92.2%)	
CG or GG	84 (9.9%)	66 (7.8%)	
Recessive Model			0.218
CC or CG	842 (99.5%)	846 (99.9%)	
GG	4 (0.5%)	1 (0.1%)	

^a^: For Hardy–Weinberg equilibrium in the control group, the *p*-value for the test was 0.491. ESRD = End stage renal disease. GG and CC: homozygous genotype of PPARG Pro12Ala. GC: heterozygous genotype of PPARG Pro12Ala.

**Table 4:** Correlation between PPARG Pro12Ala gene polymorphisms and the risk of ESRD.

Genotype	ESRD/ Total	Crude-OR (95% CI)	*p*-Value	Adj-OR ^a^ (95% CI)	*p*-Value
Allele					
C Allele	1627/3231	1.00		1.00	
G Allele	67/155	0.75 (0.54–1.04)	0.084	0.75 (0.54–1.05)	0.092
Co-dominant			0.190		0.200
CC	781/1543	1.00		1.00	
CG	65/145	0.79 (0.56–1.12)	0.183	0.80 (0.57–1.13)	0.201
GG	1/5	0.24 (0.03–2.18)	0.207	0.24 (0.03–2.16)	0.202
Dominant Model					
CC	781/1543	1.00		1.00	
CG or GG	66/150	0.77 (0.55–1.07)	0.123	0.77 (0.55–1.08)	0.135
Recessive Model					
CC or CG	846/1688	1.00		1.00	
GG	1/5	0.25 (0.03–2.23)	0.214	0.24 (0.03–2.20)	0.208

^a^: Correct sex, age.

**Table 5:** Effect of regulatory factors in the meta-analysis for Pro12Ala and CKD (alleles).

Regulatory Factor	n	τ2	Adjusted τ2	OR	95% CI	p-Value $	Egger’s Test
Race	20	0.134	0.149	0.91	0.58–1.44	0.695	0.212
Gender	15	0.155	0.103	0.04	0.00–0.67	0.025	0.793
BMI	11	0.131	0.162	1.40	0.38–5.08	0.610	0.655
Diabetes	19	0.104	0.113	0.65	0.19–2.28	0.502	0.280

Race: Asians are the reference group; Sex: Male ratio (per 100%); BMI: Mean BMI (per 10 kg/m^2^); Diabetes: prevalence of diabetes (per 100%) ^$^: *p* < 0.05/4 due to Bonferroni correction. Egger’s test: *p*-value of Egger’s test.

## Supplementary Materials

The following are available online at https://www.mdpi.com/2073-4425/11/6/705/s1, Table S1: PRISMA 2009 Checklist; Table S2: Search strategies and detailed records; Table S3: General description of publications included in the meta-analysis; Table S4: Information extracted from publications included in the meta-analysis; Table S5: The list of article references in Figure 3A; Figure S1: Forest and funnel plots for the dominant model of correlation between PPARG Pro12Ala and CKD (not including this study); Figure S2: Forest and funnel plots of correlation between PPARG Pro12Ala alleles and CKD (not including this study); Figure S3: Estimation of the Caucasian sample for the correlation between PPARG Pro12Ala and CKD; Figure S4: Forest plot and funnel plot of the dominant/recessive model for the correlation between PPARG Pro12Ala and CKD (including this study).
