# Supplementary material for: PPARG Pro12Ala Polymorphism with CKD in Asians: A Meta-Analysis Combined with a Case-Control Study—A Key for Reaching Null Association
Source: Genes (Basel). 2020 Jun 26;11(6):705. doi: 10.3390/genes11060705 (PMC7349649; doi:10.3390/genes11060705)
Supplement: Supplementary file 1 [file genes-11-00705-s001.zip › genes-826389-supplementary/S1 Figure.pdf]

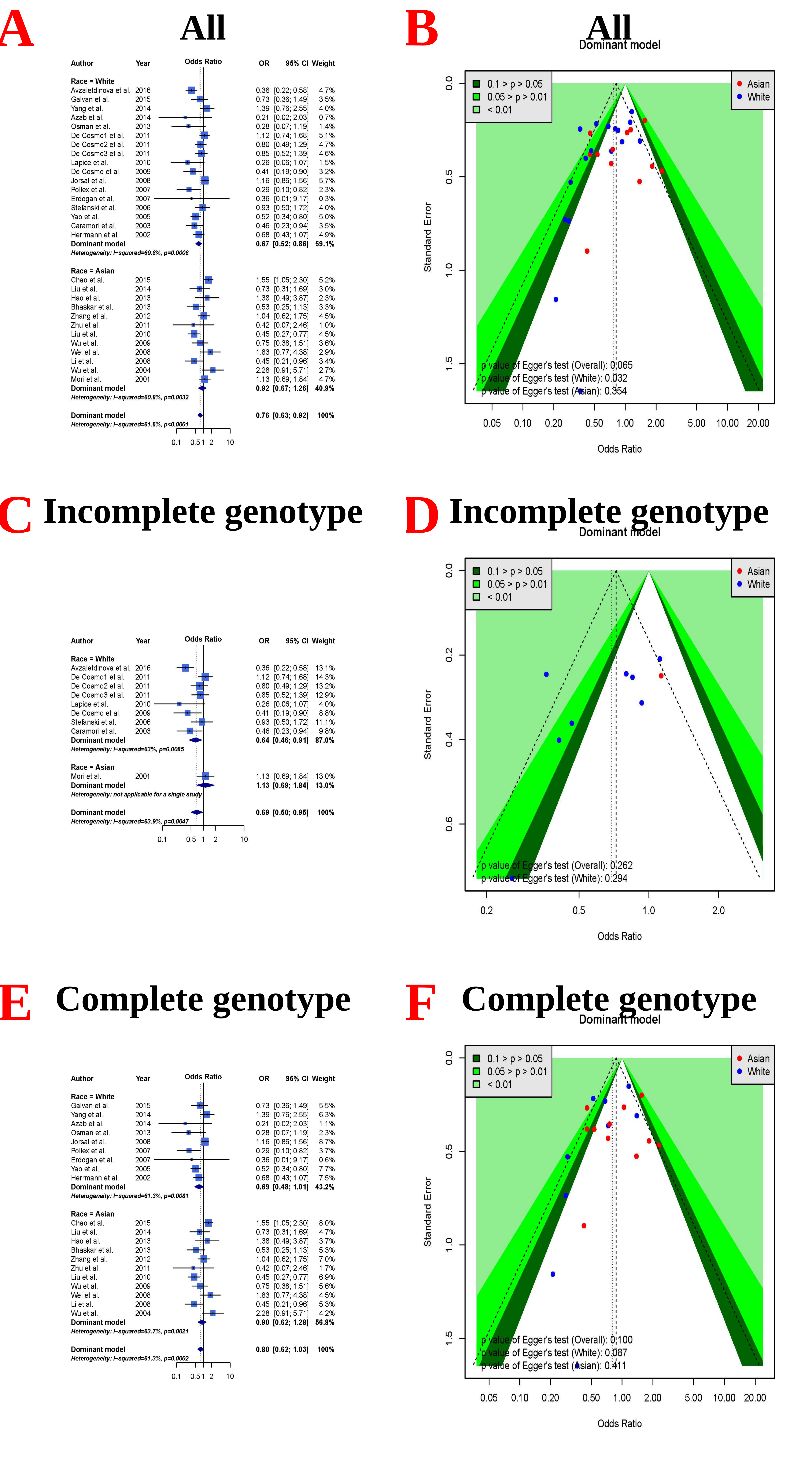
A
All
Author Year Odds Ratio OR 95% CI Weight
Race = White
Avzaletdinova et al. 2016 0.36 [0.22; 0.58] 4.7%
Galvan et al. 2015 0.73 [0.36; 1.49] 3.5%
Yang et al. 2014 1.39 [0.76; 2.55] 4.0%
Azab et al. 2014 0.21 [0.02; 2.03] 0.7%
Osman et al. 2013 0.28 [0.07; 1.19] 1.4%
De Cosmo1 et al. 2011 1.12 [0.74; 1.68] 5.1%
De Cosmo2 et al. 2011 0.80 [0.49; 1.29] 4.7%
De Cosmo3 et al. 2011 0.85 [0.52; 1.39] 4.6%
Lapice et al. 2010 0.26 [0.06; 1.07] 1.5%
De Cosmo et al. 2009 0.41 [0.19; 0.90] 3.2%
Jorsal et al. 2008 1.16 [0.86; 1.56] 5.7%
Pollex et al. 2007 0.29 [0.10; 0.82] 2.3%
Erdogan et al. 2007 0.36 [0.01; 9.17] 0.3%
Stefanski et al. 2006 0.93 [0.50; 1.72] 4.0%
Yao et al. 2005 0.52 [0.34; 0.80] 5.0%
Caramori et al. 2003 0.46 [0.23; 0.94] 3.5%
Herrmann et al. 2002 0.68 [0.43; 1.07] 4.9%
Dominant model 0.67 [0.52; 0.86] 59.1%
Heterogeneity: I−squared=60.8%, p=0.0006
Race = Asian
Chao et al. 2015 1.55 [1.05; 2.30] 5.2%
Liu et al. 2014 0.73 [0.31; 1.69] 3.0%
Hao et al. 2013 1.38 [0.49; 3.87] 2.3%
Bhaskar et al. 2013 0.53 [0.25; 1.13] 3.3%
Zhang et al. 2012 1.04 [0.62; 1.75] 4.5%
Zhu et al. 2011 0.42 [0.07; 2.46] 1.0%
Liu et al. 2010 0.45 [0.27; 0.77] 4.5%
Wu et al. 2009 0.75 [0.38; 1.51] 3.6%
Wei et al. 2008 1.83 [0.77; 4.38] 2.9%
Li et al. 2008 0.45 [0.21; 0.96] 3.4%
Wu et al. 2004 2.28 [0.91; 5.71] 2.7%
Mori et al. 2001 1.13 [0.69; 1.84] 4.7%
Dominant model 0.92 [0.67; 1.26] 40.9%
Heterogeneity: I−squared=60.8%, p=0.0032
Dominant model 0.76 [0.63; 0.92] 100%
Heterogeneity: I−squared=61.6%, p<0.0001
0.1 0.5 1 2 10
B
All
Dominant model
0.1 > p > 0.05
0.05 > p > 0.01
< 0.01
Asian
White
Standard Error
0.0 0.5 1.0 1.5
p value of Egger's test (Overall): 0.065
p value of Egger's test (White): 0.032
p value of Egger's test (Asian): 0.354
0.05 0.10 0.20 0.50 1.00 2.00 5.00 10.00 20.00
Odds Ratio
C
Incomplete genotype
Author Year Odds Ratio OR 95% CI Weight
Race = White
Avzaletdinova et al. 2016 0.36 [0.22; 0.58] 13.1%
De Cosmo1 et al. 2011 1.12 [0.74; 1.68] 14.3%
De Cosmo2 et al. 2011 0.80 [0.49; 1.29] 13.2%
De Cosmo3 et al. 2011 0.85 [0.52; 1.39] 12.9%
Lapice et al. 2010 0.26 [0.06; 1.07] 4.0%
De Cosmo et al. 2009 0.41 [0.19; 0.90] 8.8%
Stefanski et al. 2006 0.93 [0.50; 1.72] 11.1%
Caramori et al. 2003 0.46 [0.23; 0.94] 9.8%
Dominant model 0.64 [0.46; 0.91] 87.0%
Heterogeneity: I−squared=63%, p=0.0085
Race = Asian
Mori et al. 2001 1.13 [0.69; 1.84] 13.0%
Dominant model 1.13 [0.69; 1.84] 13.0%
Heterogeneity: not applicable for a single study
Dominant model 0.69 [0.50; 0.95] 100%
Heterogeneity: I−squared=63.9%, p=0.0047
0.1 0.5 1 2 10
D
Incomplete genotype
Dominant model
0.1 > p > 0.05
0.05 > p > 0.01
< 0.01
Asian
White
Standard Error
0.0 0.2 0.4 0.6
p value of Egger's test (Overall): 0.262
p value of Egger's test (White): 0.294
0.2 0.5 1.0 2.0
Odds Ratio
E
Complete genotype
Author Year Odds Ratio OR 95% CI Weight
Race = White
Galvan et al. 2015 0.73 [0.36; 1.49] 5.5%
Yang et al. 2014 1.39 [0.76; 2.55] 6.3%
Azab et al. 2014 0.21 [0.02; 2.03] 1.1%
Osman et al. 2013 0.28 [0.07; 1.19] 2.3%
Jorsal et al. 2008 1.16 [0.86; 1.56] 8.7%
Pollex et al. 2007 0.29 [0.10; 0.82] 3.7%
Erdogan et al. 2007 0.36 [0.01; 9.17] 0.6%
Yao et al. 2005 0.52 [0.34; 0.80] 7.7%
Herrmann et al. 2002 0.68 [0.43; 1.07] 7.5%
Dominant model 0.69 [0.48; 1.01] 43.2%
Heterogeneity: I−squared=61.3%, p=0.0081
Race = Asian
Chao et al. 2015 1.55 [1.05; 2.30] 8.0%
Liu et al. 2014 0.73 [0.31; 1.69] 4.7%
Hao et al. 2013 1.38 [0.49; 3.87] 3.7%
Bhaskar et al. 2013 0.53 [0.25; 1.13] 5.3%
Zhang et al. 2012 1.04 [0.62; 1.75] 7.0%
Zhu et al. 2011 0.42 [0.07; 2.46] 1.7%
Liu et al. 2010 0.45 [0.27; 0.77] 6.9%
Wu et al. 2009 0.75 [0.38; 1.51] 5.6%
Wei et al. 2008 1.83 [0.77; 4.38] 4.5%
Li et al. 2008 0.45 [0.21; 0.96] 5.3%
Wu et al. 2004 2.28 [0.91; 5.71] 4.2%
Dominant model 0.90 [0.62; 1.28] 56.8%
Heterogeneity: I−squared=63.7%, p=0.0021
Dominant model 0.80 [0.62; 1.03] 100%
Heterogeneity: I−squared=61.3%, p=0.0002
0.1 0.5 1 2 10
F
Complete genotype
Dominant model
0.1 > p > 0.05
0.05 > p > 0.01
< 0.01
Asian
White
Standard Error
0.0 0.5 1.0 1.5
p value of Egger's test (Overall): 0.100
p value of Egger's test (White): 0.087
p value of Egger's test (Asian): 0.411
0.05 0.10 0.20 0.50 1.00 2.00 5.00 10.00 20.00
Odds Ratio
