# Supplementary material for: PPARG Pro12Ala Polymorphism with CKD in Asians: A Meta-Analysis Combined with a Case-Control Study—A Key for Reaching Null Association
Source: Genes (Basel). 2020 Jun 26;11(6):705. doi: 10.3390/genes11060705 (PMC7349649; doi:10.3390/genes11060705)
Supplement: Supplementary file 1 [file genes-11-00705-s001.zip › genes-826389-supplementary/S2 Table.pdf]

S2 Table. **Search strategies and detailed records.**

Relevant text of PPAR Pro12Ala

1. Peroxisome Proliferator-Activated Receptor gamma
2. PPAR gamma
3. mPPARgamma
4. mPPARgamma1
5. mPPARgamma2
6. PPARgamma
7. PPARgamma2
8. PPARgamma3
9. Thiazolidinedione Receptor
10. Pro12Ala
11. Pro12Ala
12. SNP
13. rs1801282
14. ((1 or 2 or 3 or 4 or 5 or 6 or 7 or 8 or 9) and ((10 or 11 or 12)) or 13

Relevant text of chronic kidney disease

15. Renal Insufficiency, Chronic
16. Chronic kidney disease
17. Chronic Kidney Insufficiency
18. Chronic Renal Diseases
19. Chronic Renal Insufficiency
20. Kidney Insufficiency, Chronic
21. CKD
22. Kidney Failure, Chronic
23. Chronic Kidney Failure
24. End-Stage Kidney Disease
25. End-Stage Renal Disease
26. End-Stage Renal Failure
27. Renal Disease, End-Stage
28. Renal Failure, Chronic
29. Renal Failure, End-Stage
30. ESKD
31. ESRD
32. ESRF
33. Proteinuria
34. Albuminuria
35. Nephropathy
36. Nephritis
37. Dialysis
38. Glomerular filtration rate
39. GFR
40. 15 or 16 or 17 or 18 or 19 or 20 or 21 or 22 or 23 or 24 or 25 or 26 or 27 or 28 or 29 or 30 or 31 or 32 or 33 or 34 or 35 or 36 or 37 or 38 or 39

Combined (Final strategy)

41. 14 and 40

Web sites and uniform resource locator:

PUBMED: http://www.ncbi.nlm.nih.gov/pubmed

EMBASE: https://www.embase.com

Records from PUBMED [1-36]

Mata-analysis [1-6], Comment or response [7], No appropriate controlled group [8-10], Duplicated sample [11], Age < 18 [12], Inconsistent CKD definition [13-17], Inconsistent SNP definition [18-20], Included studies [21-36]

Records from EMBASE [37-44]

Mata-analysis [37]**,** Inconsistent CKD definition [38-40]**,** Inconsistent SNP definition [41], No appropriate controlled group [42], Included studies [43, 44]

Reference

1. Li, T., et al., *The association between lipid metabolism gene polymorphisms and nephropathy in type 2 diabetes: a meta-analysis.* Int Urol Nephrol, 2015. **47**(1): p. 117-30.
2. Ding, J., et al., *Peroxisome proliferator-activated receptor gamma Pro12Ala polymorphism decrease the risk of diabetic nephropathy in type 2 diabetes: a meta analysis.* Int J Clin Exp Med, 2015. **8**(5): p. 7655-60.
3. Zhou, T.B., X.F. Guo, and S.S. Yin, *Association of peroxisome proliferator-activated receptor gamma Pro12Ala gene polymorphism with type 2 diabetic nephropathy risk in Caucasian population.* J Recept Signal Transduct Res, 2014. **34**(3): p. 180-4.
4. Liu, G., et al., *Relationship between PPARgamma Pro12Ala gene polymorphism and type 2 diabetic nephropathy risk in Asian population: results from a meta-analysis.* J Recept Signal Transduct Res, 2014. **34**(2): p. 131-6.
5. Wang, L., et al., *The association between the PPARgamma2 Pro12Ala polymorphism and nephropathy susceptibility in type 2 diabetes: a meta-analysis based on 9,176 subjects.* Diagn Pathol, 2013. **8**: p. 118.
6. Yu, R., H. Bo, and S. Huang, *Association between the PPARG gene polymorphism and the risk of diabetic nephropathy: a meta-analysis.* Genet Test Mol Biomarkers, 2012. **16**(5): p. 429-34.
7. Lapice, E., et al., *Comment on: Zhang et al. Peroxisome proliferator-activated receptor gamma polymorphism Pro12Ala is associated with nephropathy in type 2 diabetes: evidence from meta-analysis of 18 studies. Diabetes Care 2012;35:1388-1393.* Diabetes Care, 2013. **36**(1): p. e18.
8. Chao, C.T., et al., *Diabetes mellitus, superoxide dismutase and peroxisome proliferator activated receptor gamma polymorphisms modify the outcome of end-stage renal disease patients of Han Chinese origin.* Nephrology (Carlton), 2016.
9. Lapice, E., et al., *The PPARgamma2 Pro12Ala variant is protective against progression of nephropathy in people with type 2 diabetes.* J Transl Med, 2015. **13**: p. 85.
10. Szeto, C.C., et al., *Peroxisome proliferator-activated receptor-gamma gene polymorphism and risk of cardiovascular disease in patients with diabetic nephropathy.* Am J Nephrol, 2008. **28**(5): p. 715-22.
11. Chao, C.T., et al., *Interplay between Superoxide Dismutase, Glutathione Peroxidase, and Peroxisome Proliferator Activated Receptor Gamma Polymorphisms on the Risk of End-Stage Renal Disease among Han Chinese*

Patients. Oxid Med Cell Longev, 2016. **2016**: p. 8516748.

12. Jin, J., et al., *Correlation between PPAR Gene Polymorphisms and Primary Nephrotic Syndrome in Children.* PPAR Res, 2013. **2013**: p. 927915.
13. Hosseini, S.M., et al., *The association of previously reported polymorphisms for microvascular complications in a meta-analysis of diabetic retinopathy.* Hum Genet, 2015. **134**(2): p. 247-57.
14. Mohamed Youssef, S., et al., *Interaction Effects of the Leu162Val PPAR alpha and Pro12Ala PPAR gamma 2 Gene Variants with Renal Function in Metabolic Syndrome Population.* PPAR Res, 2013. **2013**: p. 329862.
15. Hocher, B., et al., *Interaction of maternal peroxisome proliferator-activated receptor gamma2 Pro12Ala polymorphism with fetal sex affects maternal glycemic control during pregnancy.* Pharmacogenet Genomics, 2010. **20**(2): p. 139-42.
16. Malecki, M.T., et al., *Alanine variant of the Pro12Ala polymorphism of the PPARgamma gene might be associated with decreased risk of diabetic retinopathy in type 2 diabetes.* Diabetes Res Clin Pract, 2008. **80**(1): p. 139-45.
17. Smith, W.M., et al., *Opposite association of two PPARG variants with cancer: overrepresentation of H449H in endometrial carcinoma cases and underrepresentation of P12A in renal cell carcinoma cases.* Hum Genet, 2001. **109**(2): p. 146-51.
18. Gayathri, S.B., et al., *Association of the PPARGC1A gene polymorphism with diabetic nephropathy in an Asian Indian population (CURES-41).* Metab Syndr Relat Disord, 2010. **8**(2): p. 119-26.
19. Omori, S., et al., *Association of CDKAL1, IGF2BP2, CDKN2A/B, HHEX, SLC30A8, and KCNJ11 with susceptibility to type 2 diabetes in a Japanese population.* Diabetes, 2008. **57**(3): p. 791-5.
20. Sale, M.M., et al., *Variants of the transcription factor 7-like 2 (TCF7L2) gene are associated with type 2 diabetes in an African-American population enriched for nephropathy.* Diabetes, 2007. **56**(10): p. 2638-42.
21. Zambrano-Galván, G., et al., *Risk of microalbuminuria in relatives of subjects with diabetic nephropathy: A predictive model based on multivariable dimensionality reduction approach.* Clinical Nephrology, 2015. **83**(2): p. 86-92.
22. Chao, C.T., et al., *Sequence variants of peroxisome proliferator-activated receptor-gamma gene and the clinical courses of patients with end-stage renal disease.* Dis Markers, 2015. **2015**: p. 763459.
23. Yang, B., et al., *The Rate of Decline of Glomerular Filtration Rate May Not*

Be Associated with Polymorphism of the PPAR2 Gene in Patients with Type 1 Diabetes and Nephropathy. PPAR Res, 2014. **2014**: p. 523584.

24. Liu, F., et al., *Association of peroxisome proliferator-activated receptorγ gene Pro12Ala and C161T polymorphisms with cardiovascular risk factors in maintenance hemodialysis patients.* Molecular Biology Reports, 2014. **41**(11): p. 7555-7565.
25. Azab, M.M., et al., *Peroxisome proliferator activated receptor gamma2 gene Pro12Ala gene polymorphism in type 2 diabetes and its relationship with diabetic nephropathy.* Clin Lab, 2014. **60**(5): p. 743-9.
26. Bhaskar, L.V., et al., *Role of the ACE ID and PPARG P12A Polymorphisms in Genetic Susceptibility of Diabetic Nephropathy in a South Indian Population.* Nephrourol Mon, 2013. **5**(3): p. 813-7.
27. Zhang, H., et al., *Peroxisome proliferator-activated receptor gamma polymorphism Pro12Ala Is associated with nephropathy in type 2 diabetes: evidence from meta-analysis of 18 studies.* Diabetes Care, 2012. **35**(6): p. 1388-93.
28. De Cosmo, S., et al., *PPARgamma2 P12A polymorphism and albuminuria in patients with type 2 diabetes: a meta-analysis of case-control studies.* Nephrol Dial Transplant, 2011. **26**(12): p. 4011-6.
29. Liu, L., et al., *Pro12Ala polymorphism in the PPARG gene contributes to the development of diabetic nephropathy in Chinese type 2 diabetic patients.* Diabetes Care, 2010. **33**(1): p. 144-9.
30. Lapice, E., et al., *Pro12Ala polymorphism in the PPARG gene contributes to the development of diabetic nephropathy in Chinese type 2 diabetic patients: comment on the study by Liu et al.* Diabetes Care, 2010. **33**(8): p. e114; author reply e115.
31. De Cosmo, S., et al., *Impact of the PPAR-gamma2 Pro12Ala polymorphism and ACE inhibitor therapy on new-onset microalbuminuria in type 2 diabetes: evidence from BENEDICT.* Diabetes, 2009. **58**(12): p. 2920-9.
32. Jorsal, A., et al., *The PPAR gamma 2 Pro12Ala variant predicts ESRD and mortality in patients with type 1 diabetes and diabetic nephropathy.* Mol Genet Metab, 2008. **94**(3): p. 347-51.
33. Pollex, R.L., et al., *Peroxisome proliferator-activated receptor gamma polymorphism Pro12Ala is associated with nephropathy in type 2 diabetes.* J Diabetes Complications, 2007. **21**(3): p. 166-71.
34. Yao, Q., et al., *Peroxisome proliferator-activated receptor gamma polymorphisms affect systemic inflammation and survival in end-stage renal disease patients starting renal replacement therapy.* Atherosclerosis, 2005.

182(1): p. 105-11.

35. Caramori, M.L., et al., *The human peroxisome proliferator-activated receptor gamma2 (PPARgamma2) Pro12Ala polymorphism is associated with decreased risk of diabetic nephropathy in patients with type 2 diabetes.* Diabetes, 2003. **52**(12): p. 3010-3.
36. Herrmann, S.M., et al., *Peroxisome proliferator-activated receptor-gamma2 polymorphism Pro12Ala is associated with nephropathy in type 2 diabetes: The Berlin Diabetes Mellitus (BeDiaM) Study.* Diabetes, 2002. **51**(8): p. 2653-7.
37. Cao, Y., et al., *Meta-analysis of the association of Prol2Ala polymorphism of peroxisome proliferator activated receptor gamma gene with diabetic kidney disease in Chinese Han population.* Hong Kong Journal of Nephrology, 2015. **17**(2): p. S8.
38. Algenabi, A.H.A.F. and B.a.A. Hadi, *Study of PPARG2 Gene Polymorphism (Pro12Ala) in Iraqi Patients with Type 2 Diabetes Mellitus.* 2016.
39. Liu, Y.Q., et al., *Association of Pro12Ala polymorphism of PPAR-γ2 Gene and diabetic retinopathy in type 2 diabetes.* International Eye Science, 2013. **13**(6): p. 1108-1111.
40. Kuo, T.Y., et al., *A two-stage matched case-control study on multiple hypertensive candidate genes in han Chinese.* American Journal of Hypertension, 2012. **25**(7): p. 804-811.
41. Wang, S.Y., et al., *The association between FNDC5 gene polymorphisms and diabetic retinopathy in Chinese type 2 diabetes patients.* Diabetes Research and Clinical Practice, 2014. **106**: p. S89.
42. Kurzawski, M., et al., *Analysis of common type 2 diabetes mellitus genetic risk factors in new-onset diabetes after transplantation in kidney transplant patients medicated with tacrolimus.* European Journal of Clinical Pharmacology, 2012. **68**(12): p. 1587-1594.
43. Avzaletdinova, D.S., et al., *Association of variable rs1801282 locus of PPARG2 gene with diabetic nephropathy.* Russian Journal of Genetics, 2016. **52**(8): p. 877-881.
44. Osman, N.A., et al., *The association between diabetic nephropathy and polymorphisms of PPAR PRO12ALA and CCR5 32 genes in type 2 diabetes.* Nephrology Dialysis Transplantation, 2013. **28**: p. i382-i383.
