# Supplementary material for: PPARG Pro12Ala Polymorphism with CKD in Asians: A Meta-Analysis Combined with a Case-Control Study—A Key for Reaching Null Association
Source: Genes (Basel). 2020 Jun 26;11(6):705. doi: 10.3390/genes11060705 (PMC7349649; doi:10.3390/genes11060705)
Supplement: Supplementary file 1 [file genes-11-00705-s001.zip › genes-826389-supplementary/S3 Table.pdf]

S3 Table. General description about the publications included in the meta-analysis

Author	Year	Country	Ethnicity	Study design[a]	CKD type[b]	Kidney function of case [c]	Definition of case group [d]
This Study	2017	Taiwan	Asian	CC	Mixed	ESRD	HD
Avzaletdinova	2016	Russia	Caucasian	CS	T2DN	non-ESRD	eGFR < 60ml/min/1.73m^2
Galvan	2015	Mexican	Caucasian	CS	T2DN	non-ESRD	300 > albumin ≥ 30mg/dl and UACR > 30mg/g
Chao	2015	Taiwan	Asian	CC	Mixed	ESRD	RRT
Yang	2014	England	Caucasian	CC	T1DN	non-ESRD	Albuminuria > 300mg/24hr
Liu	2014	China	Asian	CC	Mixed	ESRD	HD
Azab	2014	Egypt	Caucasian	CC	T2DN	non-ESRD	Micro-albumin
Hao	2013	China	Asian	CC	T2DN	non-ESRD	UAE > 20g/min
Osman	2013	Egypt	Caucasian	CC	T2DN	non-ESRD	Proteinuria and eGFR < 60ml/min/1.73m^2
Bhaskar	2013	India	Asian	CC	T2DN	non-ESRD	UAE > 300mg/l
Zhang	2012	India	Asian	CC	T2DN	non-ESRD	ACR >300μg/mg
Zhu	2011	China	Asian	CC	T2DN	non-ESRD	UAER ≥ 20μg/min
De Cosmo(1)	2011	Italian	Caucasian	CC	T2DN	non-ESRD	ACR > 2.5(3.5)mg/mmol in men(women)
De Cosmo(2)	2011	Italian	Caucasian	CC	T2DN	non-ESRD	ACR > 2.5(3.5)mg/mmol in men(women)
De Cosmo(3)	2011	Italian	Caucasian	CC	T2DN	non-ESRD	ACR > 2.5(3.5)mg/mmol in men(women)
Liu	2010	China	Asian	CC	T2DN	non-ESRD	AER≥30mg/24hr
Lapice	2010	Italian	Caucasian	CC	T2DN	non-ESRD	eGFR < 60ml/min/1.73m^2 or Scr > 1.2mg/dl
Wu	2009	Taiwan	Asian	CC	T2DN	non-ESRD	ACR ≥ 300mg/24h and SCr >1.7 mg/dl

Author	Year	Country	Ethnicity	Study design[a]	CKD type[b]	Kidney function of case [c]	Definition of case group [d]
De Cosmo	2009	Italian	Caucasian	CC	T2DN	non-ESRD	200 > UAE ≥ 20μg/min
Wei	2008	China	Asian	CC	T2DN	non-ESRD	Ccr < 120 ml/min/1.73m^2
Li	2008	China	Asian	CC	T2DN	non-ESRD	AER ≥ 30mg/24hr
Jorsal	2008	Danmark	Caucasian	CC	T1DN	ESRD	Albuminuria > 300mg/24hr
Pollex	2007	Canada	Caucasian	CS	T2DN	non-ESRD	ACR > 2.0(2.8)mg/mmol in men(women)
Erdogan	2007	Turk	Caucasian	CC	T2DN	non-ESRD	300 > AER ≥ 30 mg/24hr
Stefanski	2006	Poland	Caucasian	CC	T2DN	non-ESRD	Albuminuria > 30mg/24hr
Yao	2005	Sweden	Caucasian	CC	Mixed	ESRD	RRT
Wu	2004	China	Asian	CC	T2DN	non-ESRD	AER > 30mg/24hr
Caramori	2003	Brazil	Caucasian	CC	T2DN	non-ESRD	AER > 200 μg/min and serum creatinine > 2.0 mg/dl
Herrmann	2002	Germany	Caucasian	CC	T2DN	non-ESRD	UAE > 200 μg/min or RRT
Mori	2001	Japan	Asian	CC	T2DN	non-ESRD	UAE > 10 mg/ml

[a]: CC – case control study; CS – cross sectional study.

[b]: T1DN – Type 1 diabetic nephropathy; T2DN –Type 2 diabetic nephropathy; Mixed –Diabetic nephropathy, hypertensive nephropathy or kidney inflammation

[c]: ESRD – only ESRD patients; non-ESRD – not only ESRD patients.

[d]: ACR – albumin creatinine ratio; AER – albumin excretion rate; UAE –urinary albumin excretion rate; eGFR – estimated glomerular filtration rate; HD – hemodialysis; CCr – creatinine clearance; RRT – renal replacement therapy; CT – computed tomography; SCr – serum creatinine.
