# Supplementary material for: PPARG Pro12Ala Polymorphism with CKD in Asians: A Meta-Analysis Combined with a Case-Control Study—A Key for Reaching Null Association
Source: Genes (Basel). 2020 Jun 26;11(6):705. doi: 10.3390/genes11060705 (PMC7349649; doi:10.3390/genes11060705)
Supplement: Supplementary file 1 [file genes-11-00705-s001.zip › genes-826389-supplementary/S4 Table.pdf]

S4 Table. Extracted information from the publications included in the meta-analysis

<table>
<tr><th rowspan="2">Author</th><th rowspan="2">Year</th><th rowspan="2">Quality[a]</th><th rowspan="2">Sex[b]</th><th rowspan="2">BMI[c]</th><th rowspan="2">DN[d]</th><th colspan="3">Case group</th><th colspan="3">Control group</th></tr>
<tr><th>CC</th><th>CG</th><th>GG</th><th>CC</th><th>CG</th><th>GG</th></tr>
<tr><td>This Study</td><td>2017</td><td>9</td><td>50.80</td><td>24.66</td><td>80.3</td><td>781</td><td>65</td><td>1</td><td>762</td><td>80</td><td>4</td></tr>
<tr><td>Avzaletdinova</td><td>2016</td><td>2</td><td></td><td>30.18</td><td>100</td><td>140</td><td colspan="2">39</td><td>83</td><td colspan="2">64</td></tr>
<tr><td>Galvan</td><td>2015</td><td>4</td><td>30.00</td><td>29.70</td><td></td><td>46</td><td>22</td><td>2</td><td>35</td><td>24</td><td>1</td></tr>
<tr><td>Chao</td><td>2015</td><td>7</td><td>44.80</td><td></td><td>37.7</td><td>635</td><td>58</td><td>5</td><td>735</td><td>46</td><td>1</td></tr>
<tr><td>Yang</td><td>2014</td><td>6</td><td>44.16</td><td></td><td>100</td><td>90</td><td>25</td><td>1</td><td>125</td><td>23</td><td>3</td></tr>
<tr><td>Liu, F</td><td>2014</td><td>5</td><td>53.53</td><td>21.6</td><td>21.2</td><td>90</td><td>9</td><td>0</td><td>131</td><td>18</td><td>0</td></tr>
<tr><td>Azab</td><td>2014</td><td>4</td><td>52.00</td><td>28.00</td><td>100</td><td>25</td><td>1</td><td>0</td><td>21</td><td>4</td><td>0</td></tr>
<tr><td>Hao</td><td>2013</td><td>3</td><td>48.89</td><td></td><td>100</td><td>77</td><td>13</td><td>0</td><td>49</td><td>6</td><td>0</td></tr>
<tr><td>Osman</td><td>2013</td><td>6</td><td></td><td>30.8</td><td>100</td><td>32</td><td>5</td><td>0</td><td>9</td><td>5</td><td>0</td></tr>
<tr><td>Bhaskar</td><td>2013</td><td>4</td><td></td><td></td><td>100</td><td>37</td><td>17</td><td>0</td><td>36</td><td>31</td><td>0</td></tr>
<tr><td>Zhang</td><td>2012</td><td>4</td><td></td><td></td><td>100</td><td>113</td><td>26</td><td>2</td><td>206</td><td>48</td><td>1</td></tr>
<tr><td>Zhu</td><td>2011</td><td>6</td><td>53.65</td><td></td><td>100</td><td>39</td><td>2</td><td>0</td><td>33</td><td>4</td><td>0</td></tr>
<tr><td>De Cosmo(1)</td><td>2011</td><td>4</td><td>62.07</td><td>31.00</td><td>100</td><td>221</td><td colspan="2">40</td><td>499</td><td colspan="2">81</td></tr>
<tr><td>De Cosmo(2)</td><td>2011</td><td>4</td><td>51.57</td><td>30.3</td><td>100</td><td>224</td><td colspan="2">30</td><td>316</td><td colspan="2">53</td></tr>
<tr><td>De Cosmo(3)</td><td>2011</td><td>4</td><td>63.36</td><td>30.1</td><td>100</td><td>207</td><td colspan="2">25</td><td>422</td><td colspan="2">60</td></tr>
<tr><td>Liu</td><td>2010</td><td>6</td><td>52.82</td><td></td><td>100</td><td>499</td><td>33</td><td>0</td><td>199</td><td>29</td><td>0</td></tr>
<tr><td>Lapice</td><td>2010</td><td>4</td><td></td><td></td><td></td><td>53</td><td colspan="2">2</td><td>606</td><td colspan="2">89</td></tr>
<tr><td>Wu</td><td>2009</td><td>6</td><td>54.63</td><td>25.75</td><td>100</td><td>197</td><td>15</td><td>2</td><td>157</td><td>12</td><td>6</td></tr>
</table>

Author	Year	Quality[a]	Sex[b]	BMI[c]	DN[d]	Case group			Control group		
						CC	CG	GG	CC	CG	GG
De Cosmo	2009	7	52.63	29.06	100	86	7	856	170		
Wei	2008	4	51.22		100	68	13	1	89	10	0
Li	2008	4	49.09	25.70	100	150	15	0	77	17	0
Pollex	2007	8	38.99	30.39	100	89	6	0	52	12	0
Jorsal	2008	7	60.72	24.30	100	290	110	15	312	105	11
Erdogan	2007	3			100	43	0	0	47	1	0
Stefanski	2006	5	44.40	34.20	100	68	25		81	32	
Yao	2005	6	61.43	24.52	28.07	180	48	1	136	63	8
Wu	2004	4			100	194	25	1	102	6	0
Caramori	2003	5	63.5	27.40	100	93	11		169	43	
Herrmann	2002	6	49.44	28.06	100	154	37	6	144	55	4
Mori	2001	7	45.21	23.20	100	580	28		982	42	

[a]: Literature quality, score; [b]: Male ratio, percentage; [c]: Body Mass Index, kg/m^2; [d]: Prevalence of Diabetes mellitus, percentage.
