# Supplementary figures and images for: PPARG Pro12Ala Polymorphism with CKD in Asians: A Meta-Analysis Combined with a Case-Control Study—A Key for Reaching Null Association
Source: Genes (Basel). 2020 Jun 26;11(6):705. doi: 10.3390/genes11060705 (PMC7349649; doi:10.3390/genes11060705)

A

Forest plot

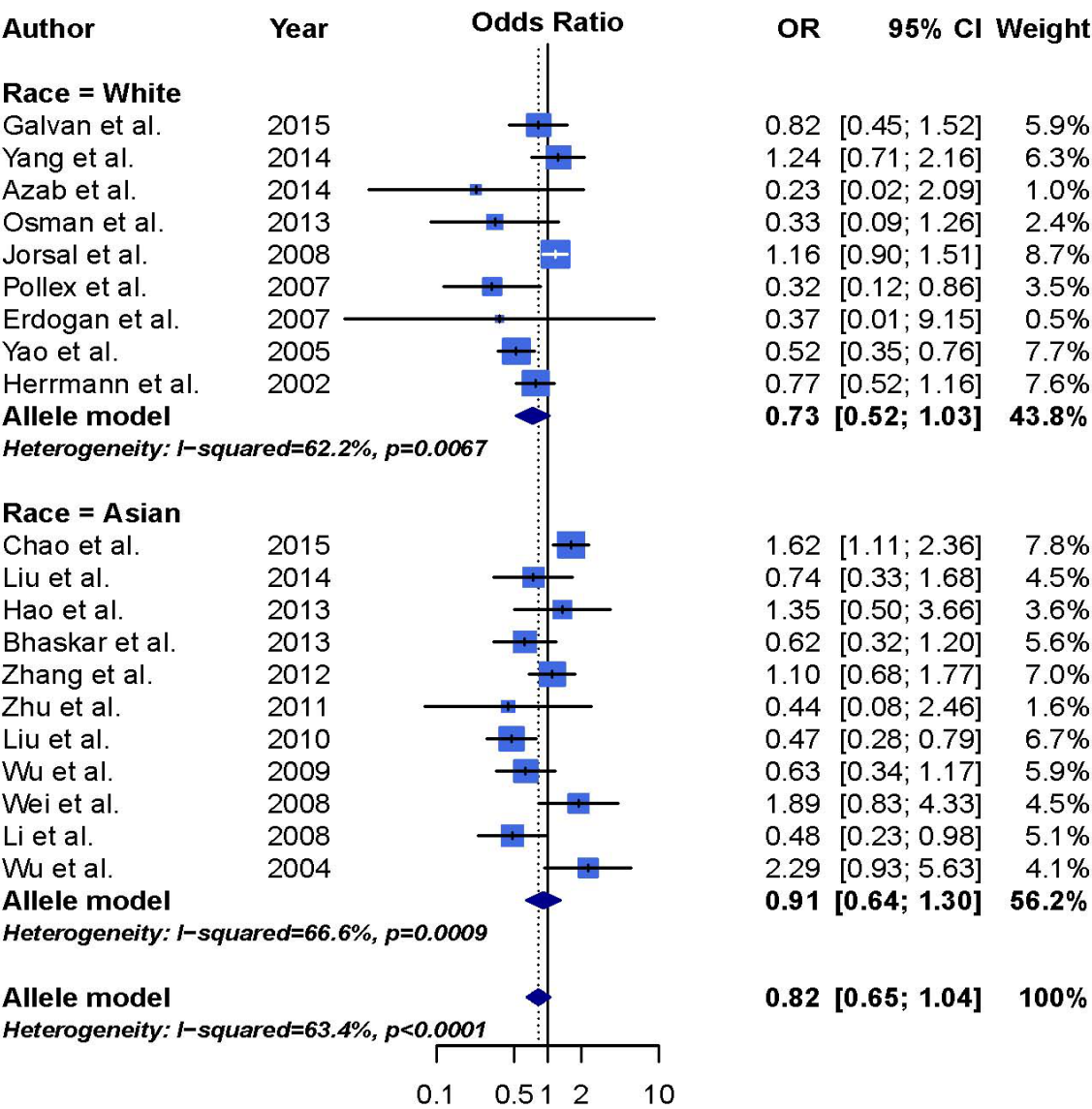

B

Funnel plot  
Allele model

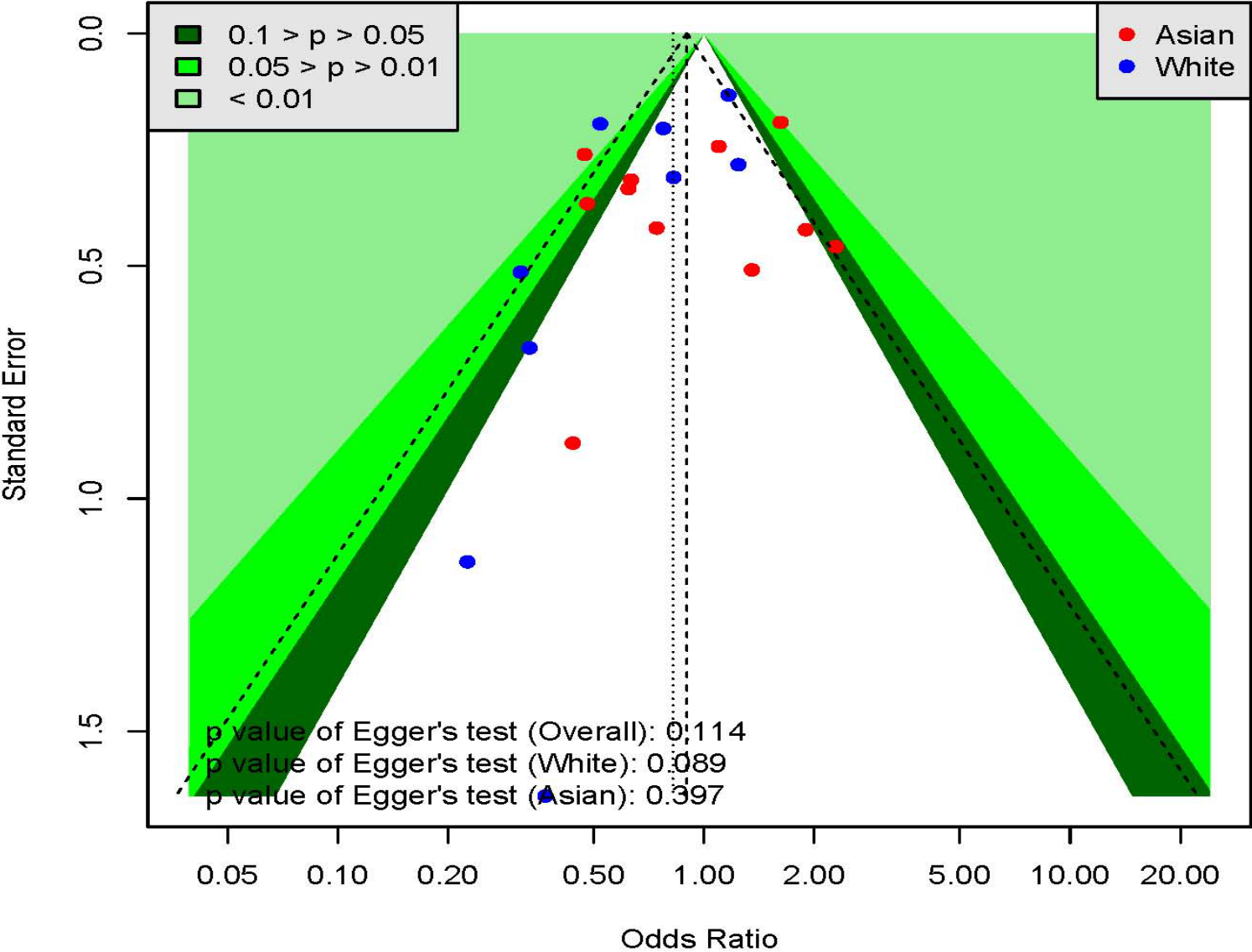

Supplement: Supplementary file 1 [file genes-11-00705-s001.zip › genes-826389-supplementary/S2 Figure.pdf]

Sample\_size is a Two-sided graph

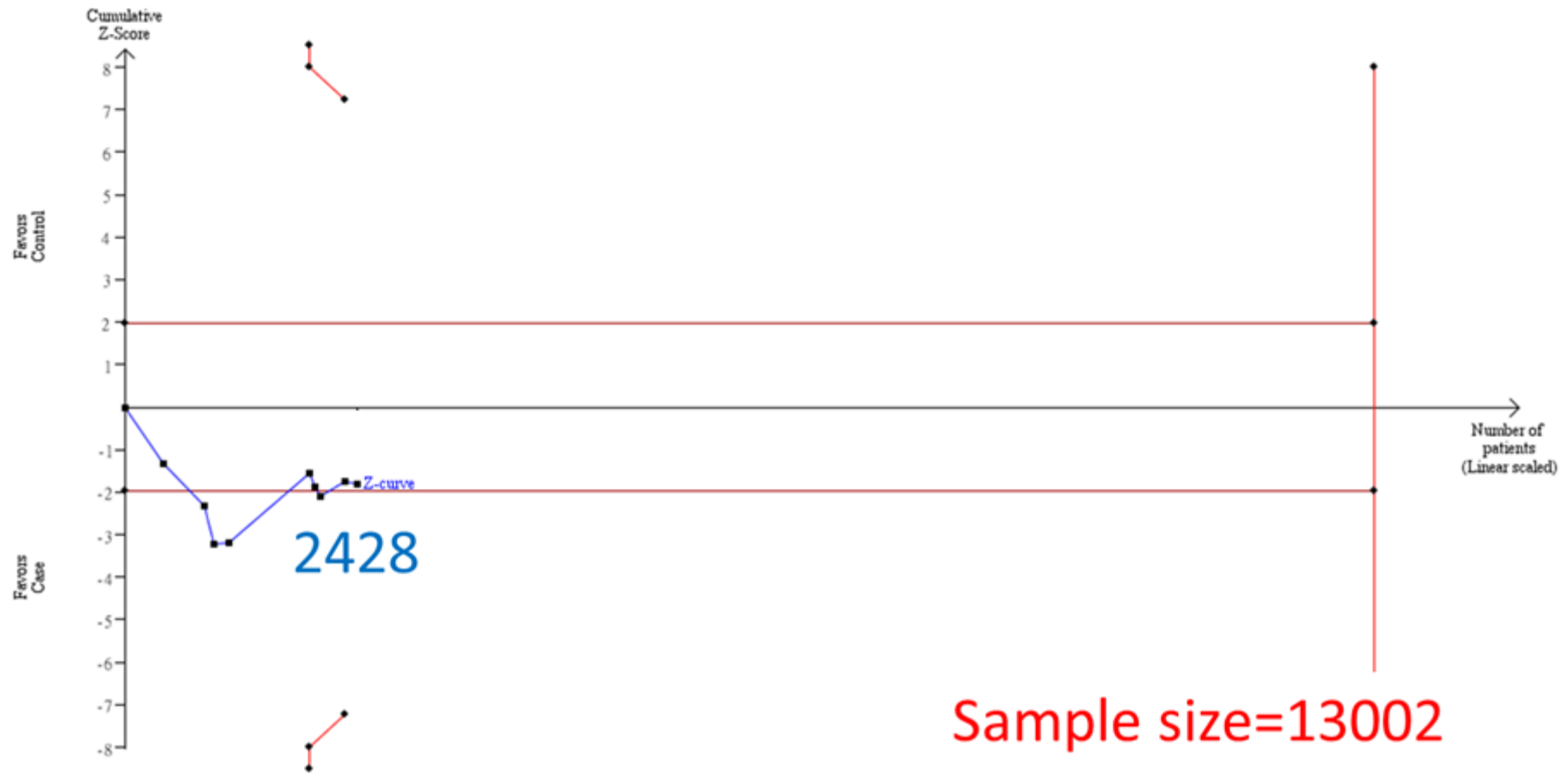

Supplement: Supplementary file 1 [file genes-11-00705-s001.zip › genes-826389-supplementary/S3 Figure.pdf]

A

# Forest plot

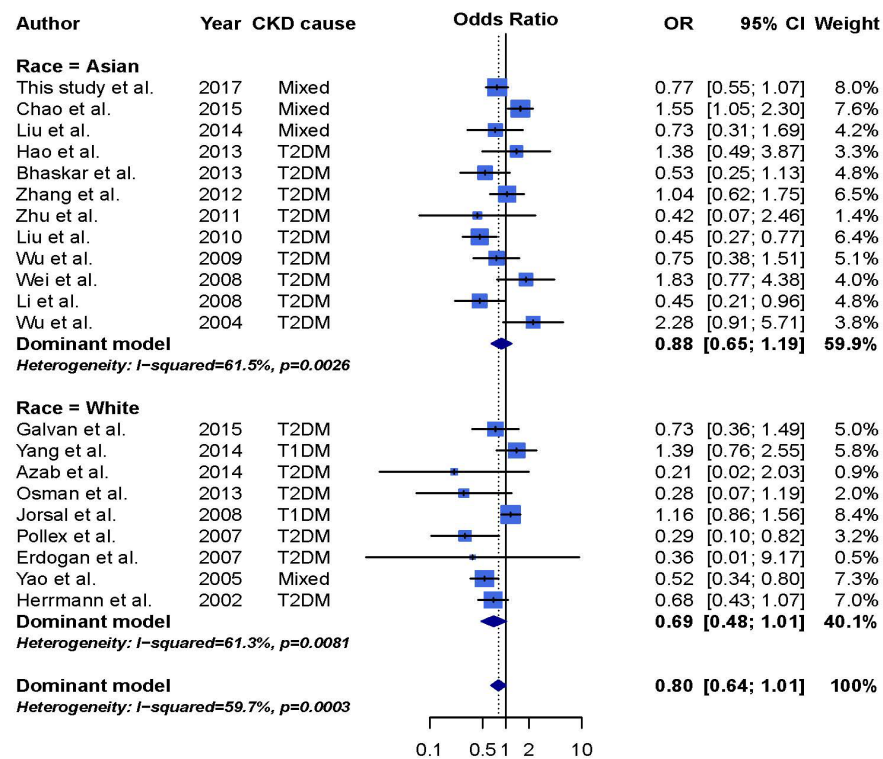

B

# Funnel plot

Dominant model

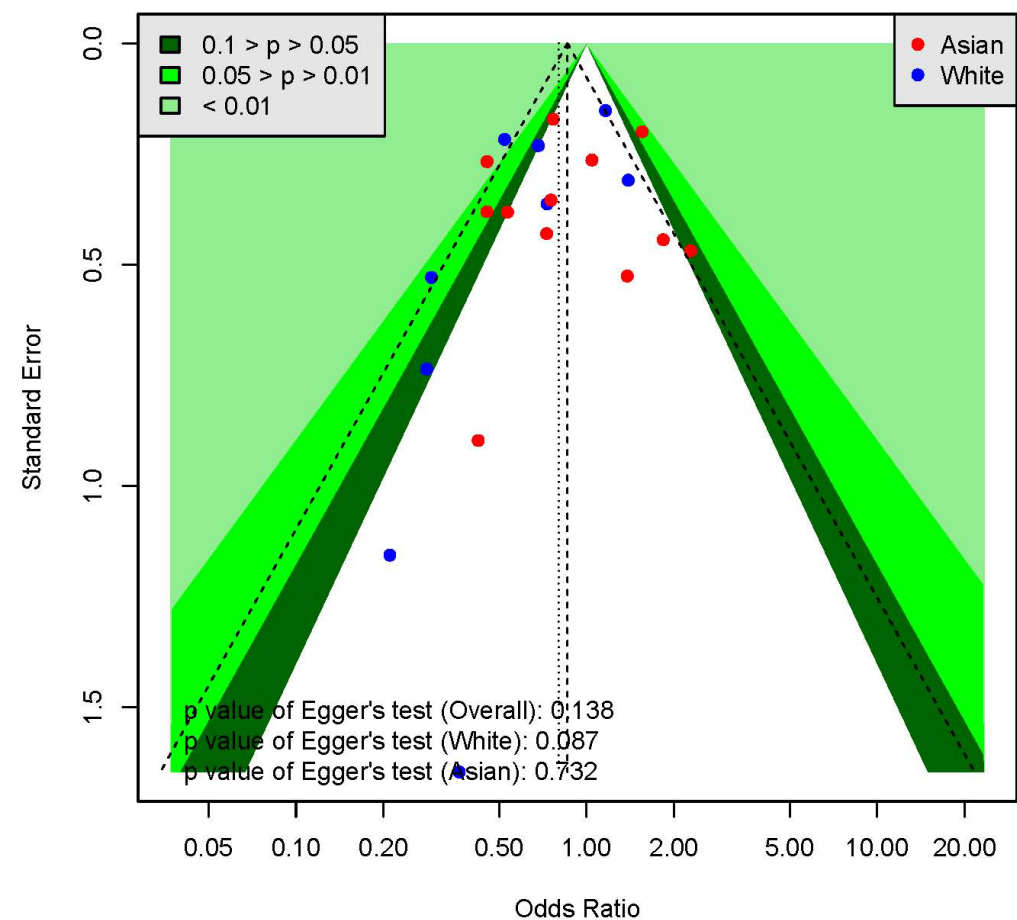

C

# Forest plot

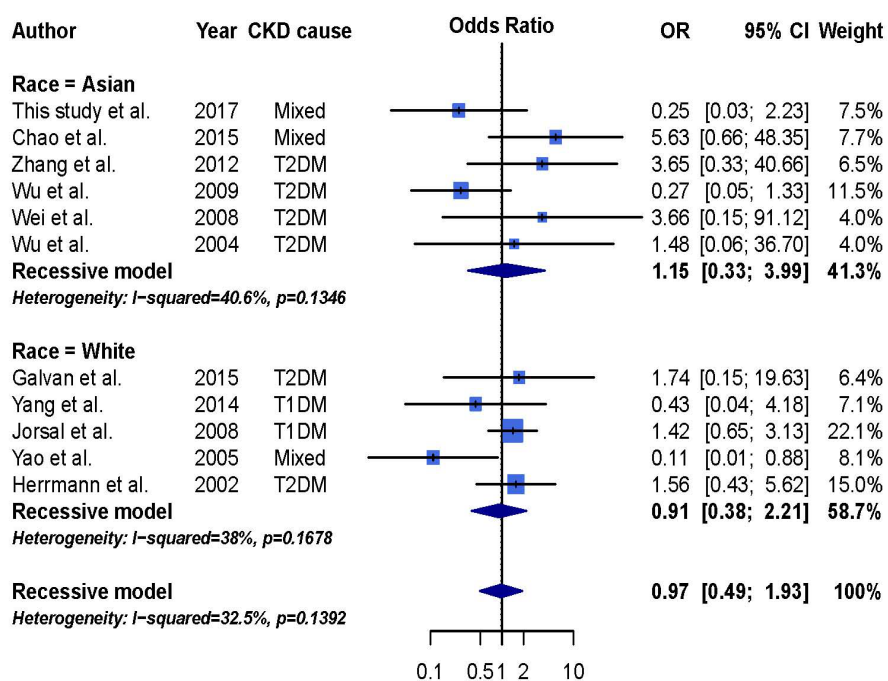

D

# Funnel plot

Recessive model

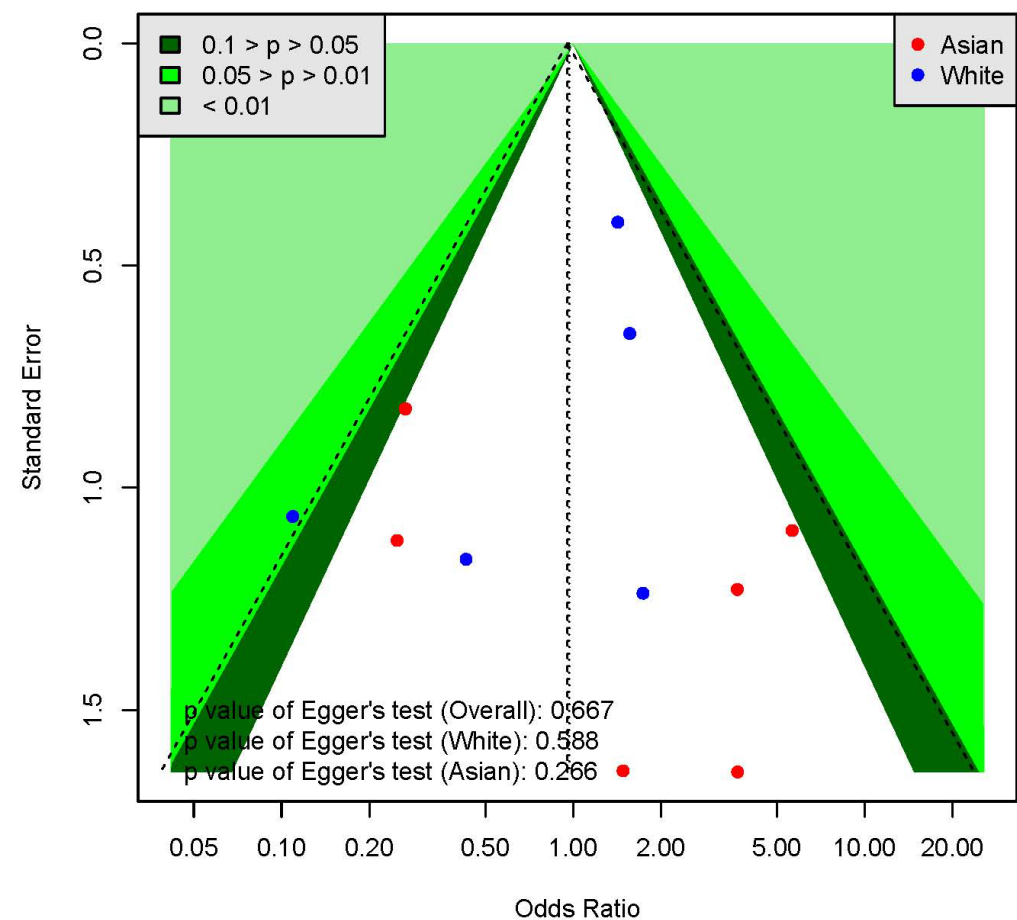

Supplement: Supplementary file 1 [file genes-11-00705-s001.zip › genes-826389-supplementary/S4 Figure.pdf]
